# Acupuncture in the treatment of infantile colic

**DOI:** 10.1186/s13052-014-0105-3

**Published:** 2015-01-15

**Authors:** Kajsa Landgren, Wolfgang Raith, Georg M Schmölzer, Holgeir Skjeie, Trygve Skonnord

**Affiliations:** Department of Health Sciences, Faculty of Medicine, Lund University, Box 157, 22100 Lund, Sweden; Division of Neonatology, Department of Paediatrics, Medical University of Graz, Graz, Austria; Research Group for Paediatric Traditional Chinese Medicine, TCM Research Center Graz, Medical University of Graz, Graz, Austria; Department of Paediatrics, University of Alberta, Edmonton, Canada; Neonatal Research Unit, Royal Alexandra Hospital, Edmonton, Alberta Health Services, Edmonton, Canada; Department of General Practice, Institute of Health and Society, University of Oslo, Oslo, Norway

**Keywords:** Infantile colic, Acupuncture, Safety

## Abstract

Regarding the recently published review ”Looking for new treatments of Infantile Colic“ by Savino et al. we want to add that positive effects of acupuncture have been demonstrated to release pain and agitation and that acupuncture seems to be a safe treatment when performed by trained acupuncturists. Inconclusive results in the few published articles on the subject can be due to different acupuncture points, different insertion time, different needling methods, differences in the outcome variables, in how the crying was measured and insufficient sample sizes. Further research is needed on understanding the utility, safety, and effectiveness of acupuncture in infants with colic.

## To the Editor

We read with interest ”Looking for new treatments of Infantile Colic“ by Savino et al [1]. This is an excellent review describing the current therapies in infantile colic. Treating infantile colic using acupuncture is feasible and without serious side effects [1], however the evidence seems to be inconclusive [1-3]. Savino et al. clearly state that there is a need for a detailed evaluation of the indications of acupuncture in newborn infants with colic, particularly during early infancy when responses are difficult to evaluate.

While we agree with the authors’ conclusion, we would like to add for the audience of the *Italian Journal of Pediatrics* and especially for centers performing acupuncture in newborn infants, that positive effects of acupuncture have been demonstrated to release pain and agitation [4]. In addition, both a recent and a 3 years older systematic review of acupuncture for children and newborns found that acupuncture is a safe treatment when performed by trained and licensed acupuncturists [4,5].

The current evidence includes three previous randomized trials [2,3,6,7] and a case series with 913 newborn infants included [8], investigating acupuncture treatment in infantile colic. Landgren et al [2,6] used two seconds of unilateral needling at acupuncture point LI4 (Large Intestine 4), and Reinthal et al [7,8] used bilateral needling for 10–20 seconds at LI4. Both reported a reduction of crying frequency and intensity in the acupuncture group compared to the control group [2,7]. A third study by Skjeie et al [3] used a different acupuncture point, bilateral needling of ST36 (Stomach 36) for 30 seconds, which is in accordance with the Norwegian Society of Medical Acupuncture, and reported no statistically significant difference in reduction of crying time between the acupuncture and the control group. These three trials [2,3,6,7] used different acupuncture points, different insertion time, and different needling methods, which potentially contributed to the inconclusive results. Other contributing factors to the variable results can be differences in the outcome variables, in how the crying was measured and insufficient sample size. For example Skjeie et al. recruited 79 patients of the 120 who were requested in the power analysis. Furthermore we want to add, that everybody who is performing paediatric acupuncture should have had adequate training and experience in acupuncture of newborn infants and children. We need future studies, recruiting larger population with feasible placebo control, to better understand the utility, safety, and effectiveness of acupuncture in newborn infants with colic.

## Abbreviations

LI4: the acupuncture point “Large Intestine 4”
ST36: the acupuncture point “Stomach 36”
